# *Pneumocystis jirovecii* detection in asymptomatic patients: what does its natural history tell us?

**DOI:** 10.12688/f1000research.10619.1

**Published:** 2017-05-23

**Authors:** Alexandre Alanio, Stéphane Bretagne

**Affiliations:** 1 Parasitology-Mycology Laboratory, Lariboisière Saint-Louis Fernand Widal Hospitals, Assistance Publique-Hôpitaux de Paris, Paris, France; 2 Paris-Diderot, Sorbonne Paris Cité University, Paris, France; 3 Molecular Mycology Unit, CNRS, Institut Pasteur, URA 3012, Paris, France

**Keywords:** Pneumocystis jirovecii, Pneumocystis pneumonia, PCP, fungal infections

## Abstract

*Pneumocystis jirovecii* is an unusual ascomycetous fungus that can be detected in the lungs of healthy individuals. Transmission from human to human is one of its main characteristics in comparison with other fungi responsible for invasive infections. *P. jirovecii* is transmitted through the air between healthy individuals, who are considered to be the natural reservoir, at least transiently. In immunocompromised patients, *P. jirovecii* multiplies, leading to subacute infections and acute life-threatening pneumonia, called Pneumocystis pneumonia [PCP]. PCP is caused by genotypically distinct mixtures of organisms in more than 90% of cases, reinforcing the hypothesis that there is constant inhalation of *P. jirovecii* from different contacts over time, although reactivation of latent organisms from previous exposures may be possible. Detection of *P. jirovecii* DNA without any symptoms or related radiological signs has been called “colonization”. This situation could be considered as the result of recent exposure to *P. jirovecii* that could evolve towards PCP, raising the issue of cotrimoxazole prophylaxis for at-risk quantitative polymerase chain reaction (qPCR)-positive immunocompromised patients. The more accurate way to diagnose PCP is the use of real-time quantitative PCR, which prevents amplicon contamination and allows determination of the fungal load that is mandatory to interpret the qPCR results and manage the patient appropriately. The detection of *P. jirovecii* in respiratory samples of immunocompromised patients should be considered for potential risk of developing PCP. Many challenges still need to be addressed, including a better description of transmission, characterization of organisms present at low level, and prevention of environmental exposure during immunodepression.

## An obligate organism of humans with a parasitic behavior

*Pneumocystis* species are fungal organisms that belong to the ascomycetous fungi based on phylogenetic studies on ribosomal ^1, 2^ and mitochondrial ^3^ DNA. These initial findings have been recently confirmed based on whole genome analysis ^4, 5^. Its closest phylogenetic organism is the plant pathogen *Taphrina deformans* ^6^. *Pneumocystis* spp. have been found in the lungs of most terrestrial mammals, and are considered to be specific to their host ^7^. Five species of *Pneumocystis* associated with specific hosts have been formally described so far: *Pneumocystis murina* in mice, *Pneumocystis carinii* and *Pneumocystis wakefieldae* in rats, *Pneumocystis oryctolagi* in rabbits, and *Pneumocystis jirovecii* in humans ^7^. It is important to note that most of the biological aspects discussed for *P. jirovecii* have actually been inferred from studies in other *Pneumocystis* species. *Pneumocystis* thrives at the surface of type I pneumocytes in the alveolar space ^8^ and is found as two main life cycle stages: the trophic form and the asci form (previously called “cysts”). Trophic forms represent the large majority of the whole *Pneumocystis* population and are thought to multiply through asexual binary fission ^8, 9^. Asci are presumably formed through the conjugation of two opposite mating type trophic forms via sexual reproduction, allowing the production of eight ascospore after maturation ^10^.

Three genomes of *Pneumocystis* spp. are available, with that of *P. jirovecii* recently released as 356 contigs ^4^. This makes comparative genomics studies possible, which are useful to understand how *Pneumocystis* evolved. One hallmark of *Pneumocystis* genomes is the lack of enzyme involved in chitin synthesis or degradation ^5^, although a cell wall is observed in asci, suggesting that it may not contain chitin, which seems rare in the fungal kingdom ^11^. In contrast, beta-glucan is present in the cell wall of asci as in other fungi ^5^. In addition, *Pneumocystis* seems to have lost thousands of genes involved in the biosynthesis of amino acids, lipids, thiamine, coenzyme A, *myo*-inositol, and biotin, assimilation of inorganic nitrogen and sulfur, and degradation of purines compared to *Taphrina deformans* ^5, 12, 13^. In contrast, proteases, RNA-processing proteins, and more specifically major surface glycoproteins (MSGs), unique to this genus, are expanded in the genome compared to other related fungal species ^5^. Interestingly, variation of the MSGs is known to induce changes in the surface antigens involved in the interface with host cells ^14^, which may facilitate evasion of the host immune response ^15^.

In total, these characteristics support the notion that *P. jirovecii* is a fungus that infects humans and exhibits parasitic behavior, meaning that *P. jirovecii* needs host nutrients to live and proliferate in the human respiratory tract. This hypothesis is reinforced by the current difficulties in cultivating *Pneumocystis in vitro*. Though low rates of multiplication of *Pneumocystis* have been reported using host cell and cell-free systems ^16– 19^, there is no *in vitro* culture that sustains the continuous growth of any *Pneumocystis* species.

## Pathophysiology of *Pneumocystis* *jirovecii* pneumonia

*P. jirovecii* is responsible for a form of acute lung injury called PCP, which occurs mainly in immunocompromised patients, including those with HIV, and in non-HIV immunocompromised patients, such as those who have undergone solid organ transplantation, have hematological malignancies, or are receiving high-dose steroids ^20, 21^. The first description of PCP was described in cohorts of malnourished or premature infants by Vanek and Jirovec in the 1950’s ^22, 23^, initially establishing it mainly as a pediatric infection, which is not the case anymore. Overall, the pathology of *Pneumocystis* has been studied in animal models with animal-specific *Pneumocystis* species, with many findings secondarily translated into human pathology ^24^. In humans, genotyping of *P. jirovecii* is the main tool used to assess and study the epidemiology and the natural history of *P. jirovecii* infection with a specific focus on transmission ^25^.

### Transmission

The hypothesis of an environmental reservoir of *Pneumocystis* has been investigated and is considered as very unlikely ^24, 26^. On the contrary, the transmission of *Pneumocystis* has long been understood to be between individuals via air from experiments performed in immunocompromised rats or nude mice ^27, 28^ with *Pneumocystis* DNA detected in the surrounding air of infected immunosuppressed or immunocompetent animals ^29– 31^. The same evidence has also been found in humans with the observation of *P. jirovecii* DNA detection in the surrounding air of patients or healthcare workers harboring *P. jirovecii* in their respiratory tract ^32– 35^, making airborne transmission the most likely route of acquisition in humans.

The airborne transmission of *P. murina* probably occurs through acquisition of the asci form of these fungi. Indeed, echinocandin-treated mice failed to transmit the disease because echinocandin treatment had decreased the number of cysts ^36^. A 12-hour contact was sufficient to allow transmission from rats infected with asci but not those infected only with trophic forms ^37^. Moreover, immunocompetent mice exposed to *Pneumocystis*-positive immunocompromised mice harbor and carry *Pneumocystis* after multiplication, although few respiratory symptoms (mild or asymptomatic infection) could be observed ^38, 39^. In addition, the *Pneumocystis*-exposed immunocompetent mice are able to transmit the disease to *Pneumocystis*-naïve immunocompetent mice ^38, 39^. Immunocompetent mice were able to resolve infection within 5 to 6 weeks after mild symptoms ^40^. This has also been observed in immunocompetent rabbits, with clearance of *P. oryctolagi* in about 90 days with an increase of anti- *Pneumocystis* IgG from day 21 to day 90 ^41^. Together, these observations support the contention that immunocompetent hosts are likely to be a source of infection within 2 to 3 months after inoculation. Data obtained in humans corroborate this hypothesis with detection and modification of antibody titers over time of specific anti *-P. jirovecii* antibodies in healthy individuals ^15, 42^. Indeed, a serological survey of anti- *P. jirovecii* antibodies in 107 healthy infants showed a very rapid and early exposure to *P. jirovecii* over time with a maximum seroconversion rate (about 90%) at 18 months of life ^43^. Of note, *P. jirovecii* DNA had been detected in parallel in the respiratory tract of some of these infants as well ^43^. In addition, *P. jirovecii* has been found to be highly prevalent in immunocompetent individuals in a cohort of infants with sudden unexpected death ^44^ or adults who died from violent causes based on lung tissue sample analysis ^45^. Detection of *P. jirovecii* alone in immunocompetent infants with bronchiolitis supports the possibility that *P. jirovecii* could be associated with spontaneously resolved symptoms in infants ^46^. An increased incidence of *P. jirovecii* detection has been observed in pregnant women known to be physiologically immunosuppressed to some extent. This could reflect an increased exposure by older children within the family or a decreased immune control of *P. jirovecii*. Thus, pregnant women could be an important part of the reservoir of *P. jirovecii* ^47^.

**Figure 1.**
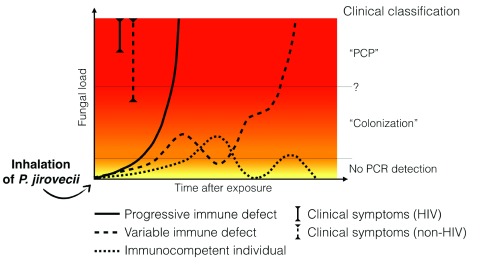
The variable evolution of *Pneumocystis* fungal load after exposure depending on the host’s immune status. Upon exposure, *P. jirovecii* multiplies at the surface of type I pneumocytes in the alveoli. At the very beginning of the infection, quantitative polymerase chain reaction (qPCR) may not be positive owing to very low or localized multiplication of *P. jirovecii*, even if a bronchoalveolar lavage (the most sensitive specimen) is performed. Depending on the host’s immune status, the evolution of the fungal load could differ: - Rapid and constant multiplication of *P. jirovecii* leading to *Pneumocystis* pneumonia (PCP) is typically observed in HIV patients or in patients under immunosuppressive regimens (regular line). The situation known as “PCP” is observed when a high fungal load is associated with symptoms. Note that symptoms appear for lower fungal loads in non-HIV patients compared to HIV patients. - Variable fungal load above or below the detection’s threshold is observed in patients with variable immune status with PCP occurring when immunosuppression increases. This situation is typically seen in hematology or cancer patients submitted to several courses of chemotherapy (dashed line). PCR detection in asymptomatic patients in the situation known as “colonization” could correspond to patients controlling the disease (decreasing fungal load) or to patients who will develop PCP in the near future. The threshold between PCP and “colonization” may be different in HIV-positive and HIV-negative patients. - Immunocompetent patients are potentially a reservoir of *P. jirovecii* with low multiplication rate due to rapid immune control and subsequent decrease of fungal load. Re-infection with a new genotype or reactivation could occur later on (dotted line).

These facts support the hypothesis that immunocompromised infants or adults should be a reservoir of *P. jirovecii* and that immunocompetent individuals should also be, at least more transiently, a reservoir of this organism. Thus, immunocompetent individuals should serve as sources of constant circulation of the fungal organism in the population, with the risk of transmission to potentially immunocompromised hosts ( Figure 1).

### Latent or recently acquired infection?

Regarding the hypothesis of constant airborne circulation of *P. jirovecii* and the early exposure in childhood, it is licit to hypothesize that PCP could be due to (i) the reactivation of organisms acquired in childhood, as demonstrated for other fungal diseases ^48, 49^, or (ii) the multiplication of recently acquired organisms. Reactivation of latent infection had been investigated in animal models and in humans. Animal studies in the lab suggested that reactivation occurred rarely after PCP (<25% of animals after 1 year) ^50, 51^. In humans, one study of recurrent pneumocystosis after treatment suggested new acquisition of *P. jirovecii* at the second episode ^52^. In this study, only five out of 10 patients harbored a different genetic pattern, suggesting that both newly acquired infection and reactivation may occur ^52^. However, this study used a genotyping method that is not sufficiently accurate to provide a definite answer to this question because it focused only on the diversity of one genetic marker (sequence of the mitochondrial large subunit ribosomal RNA gene) with variation of the sequence at only two positions.

Strong evidence for continuous acquisition of *P. jirovecii* comes from genotyping studies of PCP cases. Indeed, when PCP occurs, it has been shown that about 70% of the cases were composed of mixtures of organisms ^53– 55^. This proportion increased to more than 90% of cases if highly sensitive methods such as ultra-deep pyrosequencing were used ^56^. However, these mixtures can be the consequence of iterative inhalation of different genotypes over time or of a single exposure to different genotypes. Microevolution of *P. jirovecii* in a single host cannot be excluded.

The major evidence in favor of recent acquisition of *P. jirovecii* is the numerous description of outbreaks or clustered PCP cases in different settings including kidney transplant units ^57^, liver transplant units ^58^, pediatric oncology wards ^59^, hematology wards ^60– 62^, and wards of other medical specialties ^55, 63^. The detection of *P. jirovecii* DNA in the air surrounding individuals with or without active PCP and genotyping bridges the gap between the human source and the acquisition of a specific genotype from other individuals in a specific period and at a given location ^32– 34^. Transmission could also occur between healthy individuals such as healthcare workers and immunocompromised patients, as already suggested ^32, 35, 64^. Indeed, healthcare workers would be able to transmit *P. jirovecii* for quite a long time, since *P. jirovecii* was detected in specific individuals for up to 10 weeks ^64^. In immunocompromised patients, timing between exposure and disease (incubation time) has not been clearly defined. Therefore, it remains unanswered how long before PCP a possible transmission in the hospital setting may take place. Recently, transmission of a specific genotype in renal transplant recipients, hematology and cancer patients of a specific hospital have been studied over a period of 4 years. The median time between suspected exposure and PCP was 197 days (interquartile range: 57–342.5), suggesting that the incubation time of PCP is variable and can be as long as 3 months, even in hospitalized and immunocompromised individuals ^55^. However, in this study, patients harboring the outbreak genotype were more likely to be infected with a single genotype and not a mixture of genotypes ^55^. This suggests a recent acquisition and proliferation of this particular *P. jirovecii* genotype responsible for the outbreak in potentially *P. jirovecii*-naïve patients. In addition, genotypes found during PCP were more related to the place of PCP diagnosis rather than to the place of birth, reinforcing the hypothesis of temporarily related *P. jirovecii* acquisition in hospital settings ^65– 67^.

Therefore, it is conceivable that a *P. jirovecii* genotype acquired in the past could shape the host’s immune response. Indeed, immunity raised memory against the specific surface antigen of this specific genotype, resulting in equilibrium between host response and *P. jirovecii* multiplication. At this point, when other genotypes harboring other surface antigens are acquired, as already suggested ^14, 15, 68, 69^, this new organism could proliferate as long as the immune system has not raised an appropriate response to these new surface antigens.

Importantly, limitation of genotyping methods in samples harboring low fungal load, or the impossibility of following the natural history of PCP from *P. jirovecii* acquisition in childhood to PCP during immunosuppression, still prevents the acquisition of proof that reactivation of latent infection may occur in humans. However, it is likely that recent exposure to one or multiple *P. jirovecii* genotypes is a major route of infection in immunocompromised individuals. It cannot be excluded that both mechanisms could occur simultaneously: reactivation of *P. jirovecii* from old exposure and multiplication from recent *P. jirovecii* exposure. It is also still unknown if specific genotypes are more prone to give infection or more prone to proliferate compared to others or if some genotypes are more specific to a given host’s background.

## The concept of “colonization”

The concept of “colonization” or “carriage” of *P. jirovecii* has been introduced as early as the point at which *P. jirovecii* DNA is detected in patients without any symptoms, although at risk for PCP ^70^. This concept has been extensively reviewed ^71^. HIV infection, malignancies, or solid organ transplantation or immunosuppression secondary to immunosuppressive drugs including steroids increase the prevalence of *P. jirovecii* detection in asymptomatic patients. However, the prevalence of this “colonization” is very variable from one study to another because of either the PCR method used or the patient cohorts studied having different levels of immunosuppression ^71^. *P. jirovecii* DNA has also been detected in non-immunosuppressed patients, such as those with chronic lung diseases, cigarette smokers, or pregnant women whose immunity could be considered as abnormal ^71^. Most of the authors assume that “colonization” is a specific nosological category, which has been brought into opposition with PCP ^71^. In contrast, one may consider that “colonized” patients who are either immunocompetent or immunocompromised could be considered as recently exposed to *P. jirovecii* and then serve as reservoirs of the organism, at least transiently, as discussed above. Consequently, we propose that “colonization” could be considered as a situation where people have recently encountered *P. jirovecii*. In those individuals, only the immune status will predict if infection with symptoms will occur. Thus, what is called “colonization” or “asymptomatic carriage” could be the starting point for PCP in immunocompromised patients and therefore should not be neglected, whatever the scenario retained (recent acquisition, or reactivation of previous contamination). For instance, Mori *et al*. have shown that patients receiving immunosuppressive therapy for rheumatoid arthritis with *P. jirovecii* DNA detection without respiratory symptoms have developed PCP in the next 2 to 4 weeks ^72^. Upon *P. jirovecii* exposure or reactivation in immunocompromised hosts, the fungal load initially not detectable will increase until a plateau at the stage of PCP ^29^. The rapidity of this increase should vary according to the immune status and the degree of immunosuppression ( Figure 1) ^21, 73^.

Therefore, the risk of PCP following *P. jirovecii* DNA detection underlies the need for prophylaxis in different clinical situations of immunosuppression ^20, 74^. The generalization of prophylaxis to asymptomatic carriers of *P. jirovecii* should decrease the fungal load or even eradicate the fungus in these patients, leading to fewer cases of PCP in populations at risk, but also reduce transmission to other immunocompromised patients who share the same specialized wards. The expected benefit would be to dramatically decrease the probability of nosocomial transmission in immunocompromised hosts. This latter point obviously needs clinical validation. The risk of wide use of cotrimoxazole prophylaxis might be an increase in cotrimoxazole resistance. However, resistance following full-dose cotrimoxazole appears to be extremely rare ^75^.

## Practical considerations for the diagnosis of *Pneumocystis* pneumonia

### Diagnostic tools

The most sensitive tools to detect *P. jirovecii* are PCR-based assays. Real-time qPCR is the only PCR format assuming both a low to null rate of false positivity and quantification of the fungal load ^75^. Microscopy with immunofluorescence is much less sensitive than qPCR, with the frequent observation of negative immunofluorescence in samples containing high quantities of DNA ^75– 77^. Indeed, real-time PCR has been recommended for PCP diagnosis by a European panel of experts at the recent European Conference on Infection in Leukemia (ECIL) ^75^.

The quantification of DNA is of the utmost importance to evaluate the risk of PCP. Indeed, the immunosuppression background influences the fungal load associated with PCP ^21, 78, 79^. HIV-positive patients harbor much higher fungal loads and have a much lower mortality rate (17–30%) ^80^. In contrast, HIV-negative patients harbor lower fungal loads associated with a higher mortality rate (28–58%) ^80^. This strongly suggests that the host’s immune status is one of the most important determinants that shape the ability to control PCP and the clinical presentation ^21, 80^. Consequently, a qPCR result must provide quantification, which is to be interpreted according to the underlying diseases and the presence of risk factors ( Figure 1).

Full-blown PCP with high fungal loads with or without microscopic evidence of cysts (high fungal load) does not raise any therapeutic hesitation. The detection of *P. jirovecii* DNA, whatever the fungal load, in symptomatic immunocompromised patients should be carefully considered to be responsible, at least partially, for the symptoms and be treated accordingly ^75^. The detection of low *P. jirovecii* loads in the respiratory tract of asymptomatic patients with ongoing or scheduled immunosuppressive therapy makes the patient at risk of developing PCP in the near future and at least prophylaxis should be considered. Abstention might be advised only in non-immunocompromised patients. However, even in non-immunocompromised patients, such as those with COPD, *P. jirovecii* can play a role in the development of COPD ^81, 82^ or at least in the severity of pulmonary symptoms ^83, 84^.

Some authors have proposed using a combination of qPCR results together with beta-glucan levels to help define PCP ^85^. In this report, it was demonstrated that a correlation existed between qPCR fungal load in respiratory samples and beta-glucan levels in serum. However, when intermediate fungal loads were detected, intermediate beta-glucan loads were observed, suggesting that beta glucan does not add more value for clinical decision making. On the other hand, pneumocystosis without the detection of glucan seems possible ^86^, although rare ^75^.

### Specimens

The DNA quantification discussed above is highly dependent on the type and the quality of the respiratory sample. The use of bronchoalveolar lavage (BAL) has been recommended as the most sensitive specimen owing to the fact that BAL is the sample where the highest fungal load is expected compared to sputa or upper respiratory specimens (URS) ^87– 89^. Consequently, BAL is the only specimen able to exclude the diagnosis of PCP in case of negative qPCR results. In contrast, the absence of *P. jirovecii* detection in URS does not exclude the diagnosis if negative. If positive PCR in URS is detected, this supports the diagnosis of PCP knowing that the corresponding fungal load in the BAL should be higher than that observed in URS ^75, 90^.

Even if BAL is the specimen where the highest fungal load is expected, attention should be paid to how BAL is performed. In an autopsy study of healthy individuals, only 43% of lung tissue specimens were PCR positive, suggesting heterogeneity of the distribution of *P. jirovecii* in the lung ^45^. Indeed, *P. jirovecii* seems to be predominant in some areas of the lungs ^91^. Thus, *P. jirovecii* seems heterogeneously distributed and clustered in specific areas ^92^, as suggested from radiological analysis of PCP cases ^93, 94^. Therefore, some authors suggested sampling several lobes during the BAL procedure to increase the sensitivity of *P. jirovecii* detection ^91^. To assess the quality of the sample, it is possible to quantify, in parallel to *P. jirovecii*, human DNA in the sample ^90^.

## Conclusion/perspectives

The concept of “colonization” as a specific state, which does not warrant treatment in opposition to PCP, seems outdated regarding the accumulated knowledge about the natural history of the disease. There is a constant back and forward evolution between the fungal load and the immune status of the patients and a constant risk of transmission between patients and healthcare staff. Therefore, the detection of *P. jirovecii* should not be discarded or considered as insignificant, even in asymptomatic patients. The risk of developing PCP for a patient should be interpreted as a function of the immune status of the patient not only at the time of diagnostic workup but also in the following days/weeks. For the best results, DNA detection should rely on qPCR assays performed on BAL, although URS can also be used for clinical decision-making if positive. Many questions remain to be addressed and should trigger future research including in the transmission and prevention of exposure in hospital settings. Isolation of patients in hospitals should be discussed, and one can imagine that, in wards taking care of immunocompromised patients, it will be recommended that patients and healthcare workers wear a mask to prevent transmission to other patients in cases of cough, which is already recommended for viral or bacterial pneumonia. One can also imagine that qPCR screening using non-invasive specimens (sputa, nasopharyngeal aspirates or swabs, oral washes) in patients and healthcare workers might help implement preventive measures such as isolation of patients and/or mask wearing ^90^. Generalized (to all patients at risk of PCP) or targeted (restricted to qPCR-positive patients upon screening) cotrimoxazole prophylaxis could also be discussed, although potential toxicity should be kept in mind. To go further in the description of transmission in humans, molecular tools able to characterize low to very low quantities of *P. jirovecii* DNA are needed. Ideally, these new tools would not be based on mitochondrial DNA, since mitochondrial heteroplasmy would prevent definite results ^56^. Loci from nuclear DNA should be more appropriate, although present as a single copy, limiting the sensitivity of their detection. Eventually, new diagnostic tools based on RNA detection and quantification allowing a more precise description of the active or resting state of *P. jirovecii* in a given patient would help clinicians in deciphering whether the patient should be considered to have PCP if the multiplication is active or whether other etiological agents should be incriminated when facing resting microorganisms. These tools are under investigation and could be available in the near future ^95^.
